# COVID-19 and mental health of pregnant women in Ceará, Brazil

**DOI:** 10.11606/s1518-8787.2021055003225

**Published:** 2021-05-21

**Authors:** Márcia Maria Tavares Machado, Hermano Alexandre Lima Rocha, Marcia C. Castro, Edgar Gomes Marques Sampaio, Francisco Ariclene Oliveira, Jordan Prazeres Freitas da Silva, Camila Machado de Aquino, Liduina de Albuquerque Rocha de Sousa, Francisco Herlanio Costa Carvalho, Elisa Rachel Pisani Altafim, Luciano Lima Correia

**Affiliations:** I Universidade Federal do Ceará Faculdade de Medicina Departamento de Saúde Coletiva FortalezaCE Brasil Universidade Federal do Ceará. Faculdade de Medicina. Departamento de Saúde Coletiva. Fortaleza, CE, Brasil; II Harvard TH Chan School of Public Health Department of Global Health and Population BostonMA USA Harvard TH Chan School of Public Health. Department of Global Health and Population. Boston, MA, USA; III Universidade Federal do Ceará Faculdade de Medicina Departamento de Saúde Materno-Infantil FortalezaCE Brasil Universidade Federal do Ceará. Faculdade de Medicina. Departamento de Saúde Materno-Infantil. Fortaleza, CE, Brasil; IV Fundação Maria Cecilia Souto Vidigal São PauloSP Brasil Fundação Maria Cecilia Souto Vidigal. São Paulo, SP, Brasil

**Keywords:** Pregnant Women, Coronavirus Infections, psychology, Mental Disorders, Social Isolation

## Abstract

**OBJECTIVE:**

To assess the perceptions of pregnant women about COVID-19 and the prevalence of common mental disorders during the implemented social distancing period.

**METHODS:**

This was an observational, cross-sectional study using digital media, of pregnant women exposed to social distancing due to the COVID-19 pandemic, in Fortaleza, Ceará, Northeastern Brazil. Common mental disorders were estimated using the modified Self-Report Questionnaire-20 (SRQ-20) scale, and the feelings towards COVID-19 were assessed using the Fear of COVID-19 scale through telephone calls made in May 2020. COX multivariate regression models were used to verify the associations.

**RESULTS:**

Of the 1,041 pregnant women, 45.7% (95%CI: 42.7–48.8) had common mental disorders (CMD). All items of the Fear of COVID-19 Scale showed a significant association with the prevalence of CMD (p < 0.001). A CMD risk gradient was observed, going from a prevalence ratio of 1.52 (95%CI: 1.13–2.04) in pregnant women with two positive items to 2.70 (95%CI: 2.08–3.51) for those with four positive items. Early gestational age and the lack of prenatal care were also associated with CMD.

**CONCLUSIONS:**

The prevalence of common mental disorders in pregnant women was high during the period of social distancing and was aggravated by negative feelings towards COVID-19.

## INTRODUCTION

The global pandemic of Coronavirus Disease-19 (COVID-19), declared by the World Health Organization (WHO) on March 11, 2020, has already surpassed 32 million cases and 984,590 deaths worldwide^1^. Official data from Brazil, as of September 25, 2020, have recorded 4,657,702 confirmed cases of COVID-19, with 139,808 deaths, resulting in a case-fatality rate of 3.0%^2^. Moreover, of the 52,335 hospitalizations due to COVID-19, 521 (1.0%) were of pregnant women, of which 36 died.

Social distancing measures were implemented in many cities in Brazil to mitigate COVID-19 expansion. These measures were associated with an increased prevalence of anxiety in pregnant women, especially during the first trimester of pregnancy^3^. Pregnancy and the postpartum period have been identified as risk factors for the development and exacerbation of mental health problems^4^. Common mental disorders (CMD) in this population group include depression, generalized anxiety disorder, panic disorder, phobias, social anxiety, obsessive-compulsive disorder and post-traumatic stress disorder^5^. The worldwide prevalence of CMD in the population reaches 29.2%. In a Brazilian study with 330 pregnant women, conducted before the coronavirus pandemic, CMD outcome had a 57.1% prevalence, being associated with marital status, gestational age, planned pregnancy and bleeding^6^.

In situations of anxiety and depression, high levels of substances such as adrenaline and dopamine are produced, both responsible for stress reactions^7^. The presence of CMD during pregnancy is associated with obstetric outcomes such as miscarriage, preterm birth and low birth weight, shorter duration of exclusive breastfeeding, growth deficit, and possible child developmental delay^4,8,9^.

Studies on the influence of the COVID-19 pandemic on the mental health of pregnant women are scarce^10^. This is the first study conducted in a low-income context, in the Northeast of Brazil, aiming at assessing perceptions of pregnant women regarding COVID-19 and its effect on the prevalence of CMD during the social distancing period and at identifying possible socioeconomic and health determinants.

## METHODS

This was an observational, cross-sectional study, conducted with pregnant women exposed to social distancing due to the COVID-19 pandemic, in the city of Fortaleza, state of Ceará, Northeastern Brazil, to assess the effects of social distancing on the mental health of these women. The recommendations established by the Strengthening the Reporting of Observational Studies in Epidemiology – STROBE^11^ – were adopted.

After approval by the National Research Ethics Committee and the acceptance of the informed consent form, all pregnant women residing in the six regions of the municipality of Fortaleza, who were subjected to social distancing measures, were considered eligible. The exclusion criteria consisted of pregnant women with a diagnosis of malformation or another severe fetal disease, and pregnant women with severe pregnancy complications that could significantly affect their mental health.

Data collection started on April 21, 47 days after the beginning of the social distancing decree, and finished on May 2, 2020. An online questionnaire with 49 closed questions created on the Google Forms platform was used. The questionnaire was distributed through a link via an instant messaging application and included questions on women’s profile, mental health (anxiety, fear), and feelings about the social distancing period. The average time to fill out the questionnaire varied from 5 to 10 minutes.

The presence of CMD was estimated by 9 of the 20 items that comprise the SRQ-20 (Self-Reporting Questionnaire)^12^, including somatic symptoms (has unpleasant sensations in the stomach, poor digestion, lack of appetite, frequent headaches), depressed mood (has been crying more than usual, has been feeling sad lately), and depressive thoughts (feels like a useless/worthless person, unable to play a useful role in their life, has lost interest in things). Nine items of the scale were used because some questions were considered a potentially risky to trigger a pre-existing depressive condition (e.g., questions about suicidal ideation, the desire to take one’s life, among others) in a scenario in which direct assistance to the respondent was not feasible. The SRQ-20 result classifies respondents into positive cases of depression when the sum of the scores is higher than 7; that is, more than 35% of positive responses. Thus, to adjust the outcome to this new application, we used the same proportion for the 9-item scale, created by separating the group into tertiles, in which the last tertile was considered to be of high risk for common mental disorders (scores higher than four).

The Fear of COVID-19 Scale^13^ instrument was used to assess negative feelings regarding COVID-19 among pregnant women (e.g., “I feel uncomfortable when thinking about Coronavirus”, “I am very afraid of Coronavirus”, “I cannot sleep because I am worried about getting the Coronavirus”, and “when I watch the news and stories about COVID-19 on social media I get nervous or anxious”). Participants were classified as having between none up to 4 negative feelings. Socioeconomic and demographic variables self-reported by pregnant women were also collected, including age, education, working pattern, participation in cash transfer programs, use of health services, family composition and conditions of pregnancy.

### Statistical Analysis

To describe the sample characteristics, absolute and relative frequencies of nominal variables were estimated, as well as mean and standard deviation of quantitative variables. In a bivariate analysis, the Chi-square test was used to verify the independence between explanatory variables and CMD. Variables that showed a descriptive level < 0.20 in the bivariate analysis were used for controlling possible confounding effects in a multivariate regression model. Cox Regression was chosen because, as compared to Poisson regression, it presented more robust results. As Cox regression was originally created for survival analysis, its use in cross-sectional data requires adjustment of variances and considering the time measurement as the same for all individuals (t =1), as was proceeded in our study^14^. Logistic regression analysis was not appropriated for this data due to the highly frequent outcome producing odds overestimation. Crude and adjusted prevalence ratios were estimated together with their respective confidence intervals. A forest plot chart was built using the Cox model measures of effect. The magnitude of the associations found at a significance level of p < 0.05 was expressed as measures of the prevalence ratio. The data were analyzed using IBM SPSS software, version 24.

### Ethical Approval

Project was approved by the Ethics Committee of the Universidade Federal do Ceará, No. 4.043.259, May 2020. The authors have obtained both informed consent and ethics committee approval for studies on volunteers. The women interviewed answered the questionnaire, sent online, during the period of social distance. Before answering the questionnaire, the consent form was made available when the interviewee answered if she accepted to participate in the research.

## RESULTS

Data were collected from 1,041 pregnant women, whose ages ranged from 16 to 48 years, with a mean age of 31.3 years. The median *per capita* income of the participants was US$ 500.26. Most participants (80.5%) reported having more than 12 years of schooling and only about 5% of them were beneficiaries of the government’s cash transfer program. Less than 33% lived in a household with more than three residents. In total, 476 (45.7%) were classified as having a CMD, and 60.6% had three or four positive questions on the anxiety scale related to COVID-19 (Table 1).

**Table 1:** Characteristics of pregnant women included in the study (n = 1,041). Fortaleza, Brazil, 2020.

Variable		n	%
Age range	Up to 35 years	837	80.4
	> 35 years	204	19.6
Median (IQR) income *per capita* (US$)		500.26 (316.96–1,009.25)	
Marital status	Married or common-law marriage	921	88.5
	Widowed/Divorced/Separated	16	1.5
	Single	104	10.0
Education	Middle School or less	7	0.7
	High School	196	18.8
	College/University	397	38.1
	Postgraduate	441	42.4
Participates in the government’s cash transfer programs		51	4.9
N. of people living with the pregnant woman	Lives alone	10	1.0
	1	341	32.8
	2	299	28.7
	3	211	20.3
	4	108	10.4
	5 or more	72	6.9
Items of the SRQ scale:	1. Do you have unpleasant sensations in your stomach?	466	44.8
	2. Do you have poor digestion (heartburn, nausea, etc.)?	753	72.3
	3. Do you have lack of appetite?	222	21.3
	4. Do you have frequent headaches?	336	32.3
	5. Have you been crying more than usual?	518	49.8
	6. Have you been feeling sad lately?	603	57.9
	7. Do you feel like a useless, worthless person?	179	17.2
	8. Do you feel incapable of playing a useful role in your life?	171	16.4
	9. Have you lost interest in things?	341	32.8
Scores of the SRQ items:	0 to 1 item	221	21.2
	2 to 3 items	344	33.0
	4 to 6 items	367	35.3
	7 to 9 items	109	10.5
Common mental disorder		476	45.7
Fear of COVID-19 Scale	0 item	75	7.2
	1 item	112	10.8
	2 items	223	21.4
	3 items	448	43.0
	4 items	183	17.6

IQR: interquartile range; SRQ: Self-Report Questionnaire.

All items selected from the Fear of COVID-19 Scale instrument showed a statistically significant association with the prevalence of CMD individually (p < 0.001), and the item showing the strongest association was “I feel uncomfortable when thinking about the Coronavirus”, with a prevalence 1.87-fold higher in pregnant women with CMDs (95%CI: 1.43–2.44). The cumulative number of positive items on the scale of negative feelings towards COVID-19 showed a CMD risk gradient as the number of positive responses increased, going from a prevalence ratio of 1.52 (95%CI: 1.13–2.04) for women who had two positive items against one or none to 2.70 (95%CI: 2.08–3.51) for those who had four positive items. The wish to talk to a professional about feelings and thoughts was another condition associated with a higher prevalence of CMD (PR = 1.95; p < 0.001) (Table 2).

**Table 2:** Common mental disorders in pregnant women, according to feelings towards COVID-19 during social distancing. Fortaleza, Brazil, 2020.

Variables	Total	Common mental disorders^a^		Crude PRR (95%CI)	p^b^

		Present	Absent		
	
		%	%		
Do I feel uncomfortable thinking about the Coronavirus?^**c**^					**< 0.001**
Yes	878	49.3	50.7	1.87 (1.43–2.44)	
No	163	26.4	73.6	1	
Am I very much afraid of the Coronavirus?^**c**^					**< 0.001**
Yes	777	49.0	51.0	1.36 (1.14–1.63)	
No	264	36.0	64.0	1	
Can’t sleep because I am worried about getting the Coronavirus?^**c**^					**< 0.001**
Yes	195	69.7	30.3	1.74 (1.53–1.96)	
No	846	40.2	59.8	1	
When watching news and stories about COVID-19 on social media, do I get nervous or anxious?^**c**^					**< 0.001**
Yes	784	51.4	48.6	1.81 (1.47 - 2.22)	
No	257	28.4	71.6	1	
Would you like to talk to a professional about your thoughts and feelings?					**< 0.001**
Yes	511	60.9	39.1	1.95 (1.69–2.26)	
No	530	31.1	68.9	1	
What is your opinion about social distancing?					**0.020**
It is not a sacrifice	92	34.8	65.2	1	
It is a necessary sacrifice	923	47.5	52.5	1.36 (1.02–1.82)	
Negative feelings towards COVID-19 (n. of “Yes” answers)					**< 0.001**
0 or 1	187	25.7	74.3	1	
2	223	39.0	61.0	1.52 (1.13–2.04)	
3	448	47.8	52.2	1.86 (1.43–2.42)	
4 positive answers	183	69.4	30.6	2.70 (2.08–3.51)	

PRR: Prevalence rate ratio.

^a^ Adapted from the SRQ20 scale.

^b^ Chi-square test.

^c^ Adapted from “Fear of COVID-19 Scale” (Ahorsu et al.^13^, 2020).

Note: Values with statistical significance are shown in bold.

Regarding demographic and socioeconomic determinants, age was associated with the prevalence of CMD, with pregnant women younger than 35 years showing a 21% higher prevalence (PR = 1.21; 95%CI: 1.01–1,45; p = 0.037). Pregnant women with 12 years or less of formal education had an 18% higher risk of CMD prevalence compared to pregnant women with higher education (PR = 1.18; 95%CI: 1.02–1.38; p = 0.039). Regarding marital status, pregnant women without a partner had a 37% higher prevalence of CMD than those with a partner (PR = 1.37; 95%CI: 1.16–1.61; p = 0.003), a finding similar to those who lived in households with four or more residents (PR=1.28; 95%CI: 1.06–1.54; p = 0.031). Finally, pregnant women that worked outside the home had an 18% lower prevalence of CMD, when compared with those who did not work (p = 0.043) (Table 3).

**Table 3:** Common mental disorders in pregnant women experiencing social distancing, according to demographic, socioeconomic, and prenatal care characteristics. Fortaleza, Brazil, 2020.

Variables	Total	Common Mental Disorders^a^		Crude PRR (95%CI)	p^b^

		Present	Absent		
	
		%	%		
Socioeconomic and demographic variables					
Neighborhood where the woman lives by HDI:					0.420
Low HDI (up to 0.49)	571	47.5	52.5	1.09 (0.88–1.34)	
Medium HDI (0.50–0.79)	317	43.2	56.8	0.99 (0.79–1.24)	
High HDI (> 0.80)	142	43.7	56.3	1	
Age ranges:					**0.037**
Up to 35 years	837	47.3	52.7	1.21 (1.01–1.45)	
> 35 years	204	39.2	60.8	1	
Level of schooling:					**0.039**
Up to 12 years of schooling	203	52.2	47.8	1.18 (1.02–1.38)	
More than 12 years of schooling	838	44.2	55.8	1.00	
Marital status:					**0.003**
With a partner	921	43.9	56.1	1	
Without a partner	120	60.0	40.0	1.37 (1.16 - 1.61)	
Participates in the government’s cash transfer program:					0.927
Yes	51	45.1	54.9	1	
No	990	45.8	54.2	1.01 (0.74–1.38)	
Works outside the home:					**0.043**
No	222	53.2	46.8	1.22 (1.05–1.41)	
Yes, before social distancing	682	43.7	56.3	1.00	
Yes, before and during social distancing	137	43.8	56.2	1 (0.81–1.23)	
Number of people living with the pregnant woman:					**0.031**
0 or 1 person	351	40.5	59.5	1	
2 or 3 people	510	47.3	52.7	1.17 (1.00–1.37)	
4 or more people	180	51.7	48.3	1.28 (1.06–1.54)	
Gestational and assistance variables					
Gestational period:					**0.005**
1^st^ trimester	190	53.7	46.3	1.32 (1.11–1.56)	
2^nd^ trimester	365	48.2	51.8	1.18 (1.02–1.38)	
3^rd^ trimester	486	40.7	59.3	1	
Prenatal appointments:					**< 0.001**
Not attending prenatal appointments	43	67.4	32.6	1.57 (1.26–1.96)	
In a basic health unit	139	56.1	43.9	1.31 (1.11–1.54)	
In a private medical office/ health insurance office	859	43.0	57.0	1	
Does someone accompany you at the prenatal appointments?					0.542
Goes alone	282	47.2	52.8	1.06 (0.91–1.23)	
Child’s father/partner	695	44.5	55.5	1	
Mother	33	54.5	45.5	1.23 (0.89–1.69)	
Other	31	51.6	48.4	1.16 (0.82–1.65)	
Did you stop having prenatal consultations during the social distancing period?					**0.017**
Yes	259	52.1	47.9	1.20 (1.04–1.38)	
No	782	43.6	56.4	1	
Do you have another child aged 1 to 6 years?					0.636
Yes	303	46.9	53.1	1.04 (0.9–1.2)	
No	738	45.3	54.7	1	
Does the child’s father live in the same house?					**0.013**
Yes	924	44.2	55.8	1	
Right now he is living in another house	43	53.5	46.5	1.21 (0.91–1.62)	
No	74	60.8	39.2	1.38 (1.13–1.68)	

PRR: Prevalence rate ratio.

^a^ Adapted from the SRQ20 scale.

^b^ Chi-square test.

Note: Values with statistical significance are shown in bold.

Regarding the gestational period and prenatal care, pregnant women who were in the 1^st^ and 2^nd^ trimesters of pregnancy had a 32% and 18% higher CMD prevalence, respectively, when compared with those in the 3^rd^ trimester (p = 0.005). A higher CMD prevalence was observed among women that had no prenatal consultation (PR = 1.57; 95%CI: 1.26–1.96; p < 0.001), whose prenatal care was interrupted (PR = 1.20; 95%CI: 1.04–1.38; p = 0.017), and who declared that the child’s father did not live in the same household (PR = 1.38; 95%CI: 1.13–1.68; p = 0.013) (Table 3).

After the multivariate adjustment, a statistically significant risk gradient adjusted for CMD was observed for pregnant women with negative feelings towards COVID-19. The more negative feelings reported, the greater the CMD prevalence, reaching an adjusted PR of 3.15 (95%CI: 2.22–4.46; p < 0.001) for those with four positive responses. Moreover, an 84% adjusted risk was observed in participants that were not undergoing prenatal care (p = 0.007) and of 32% higher among those who had consultations in public health units (p = 0.042) when compared with those who had consultations in private health units. Women in the 1^st^ trimester of pregnancy had a significantly higher CMD prevalence than those in the 3^rd^ trimester (PR = 1.43; 95%CI: 1.10–1.85; p = 0.008). A higher CMD prevalence was also found among pregnant women living with four or more people (p = 0.028), with a 36% higher adjusted risk than those living with up to one resident. Pregnant women that wished to talk to the health professional about their thoughts and feelings had a 2.3-fold higher CMD prevalence (p < 0.001). Working outside the home had a protective effect, with pregnant women in this condition showing a 21% lower CMD probability, compared to housewives (p = 0.037) (Table 4 and Figure 1).

**Table 4:** Factors associated with common mental disordersa in pregnant women during the social distancing period. Fortaleza, Brazil, 2020.

Variables	Adjusted PRR^b^	95%CI	p
Score of negative feelings towards COVID-19^**c**^:			
4 positive answers	3.15	2.22–4.46	**< 0.001**
3 positive answers	1.93	1.4–2.65	**< 0.001**
2 positive answers	1.52	1.07–2.17	**0.021**
0 or 1	1		
Number of people living with the pregnant woman:			
4 or more people	1.36	1.03–1.78	**0.028**
2 or 3 people	1.20	0.96–1.49	0.105
0 or 1 person	1		
Situation of antenatal care:			
Not attending	1.84	1.19–2.85	**0.007**
Attends at the basic health unit	1.32	1.01–1.73	**0.042**
Attends at a private/medical insurance office	1		
Works outside the home:			
Yes	0.79	0.63–0.99	**0.037**
No	1		
Gestational period:			
1^st^ trimester	1.43	1.10–1.85	**0.008**
2^nd^ trimester	1.17	0.94–1.44	0.155
3^rd^ trimester	1		
Do you wish to talk to a professional about your feelings?			
Yes	2.30	1.89–2.8	**< 0.001**
No	1		

^a^ Adapted from the SRQ20 scale.

^b^ Cox regression model, controlled for the following factors: place of residence, age range, schooling, marital status, and living or not with the child’s father.

^c^ Adapted from the Fear of COVID-19 Scale (Ahorsu et al.^13^, 2020).

Note: Values with statistical significance are shown in bold.

**Figure:**
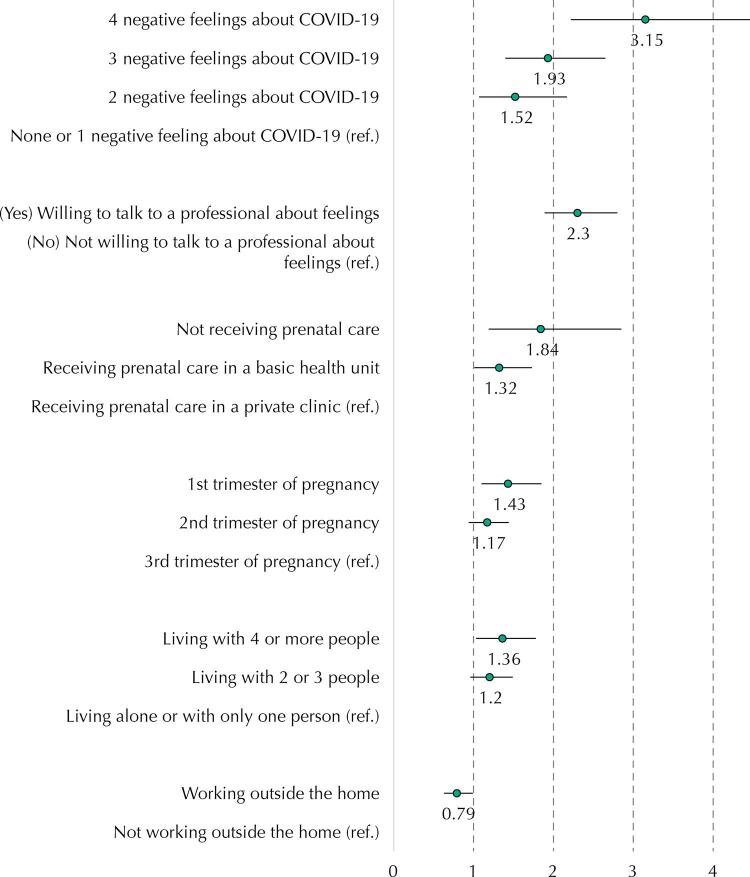
Forest plot of adjusted estimates of common mental disorders determinants in pregnant women.

## DISCUSSION

We conducted a cross-sectional study with 1,041 pregnant women in a low-income context, in the state of Ceará, Northeastern Brazil, and observed that negative feelings towards COVID-19 increase the CMD prevalence among pregnant women by three times. A higher CMD prevalence was associated with not receiving prenatal care, gestational age, and the number of residents in the household.

Our study showed a 45.7% (95%CI: 42.7–48.8) prevalence of CMD in pregnant women in Fortaleza (one of the three cities in Brazil with the highest number of COVID-19 cases, located in the impoverished Northeast region of Brazil), and all items on the Fear of COVID-19 Scale had a strong association with CMD. Although Fortaleza has a high coverage of internet access (81,4%), the CMD prevalence found can be even higher among more vulnerable mothers without internet access.

Pregnancy is a time of changes in physical, psychological, family, and social aspects that can trigger feelings of insecurity and affect the mental health of women. We observed that thinking about COVID-19 was the most important item on the fear scale, and it was associated with psychopathological symptoms, such as fear and anxiety. These feelings are expected to increase during the epidemics. For example, during the HIV, Ebola, Zika, and H1N1 outbreaks, fear increased the levels of anxiety both in healthy individuals and in those with pre-existing mental health conditions^15^. A study conducted in West Africa, in the post-Ebola epidemic, suggests that the number of individuals whose mental health is affected tends to be greater than the number of infected individuals^16^.

Our results show that watching news and stories about COVID-19 on social media and television can increase the manifestations of negative feelings in pregnant women. Seeking information during crisis events is common^17^; however, the exposure to a large number of negative news can make people feel anxious and/or distressed^18,19^. In a study on COVID-19 in India, patients recognized that after watching TV programs, listening to radio programs, and reading/watching messages on social media platforms, they developed an intense fear of becoming infected with the new coronavirus^20^. Furthermore, almost a fifth of the pregnant women in other studies reported that negative feelings towards COVID-19 have impaired their sleep, and insomnia has been previously linked to the psychopathological symptoms^21^.

The role of gestational age (1^st^ trimester of pregnancy), lack of prenatal consultation follow-up, and prenatal care (public health care network) on the CMD prevalence can be explained by two issues. First, the first months of pregnancy are marked by intense hormonal changes that affect the emotional state of the future mother^24^. Second, regular prenatal consultations have been interrupted because both the public and private health care networks are overloaded with care to patients with COVID-19^25^.

The effects of maternal age (< 35 years), education, marital status (not having a partner), number of people in the household (living with four or more people), and not working outside the home during quarantine corroborate previous findings that showed the influence of socioeconomic factors on CMD prevalence^6^.

Our results have important clinical implications, since there is an increased obstetric risk among pregnant women with CMD^23^. Therefore, in acute cases of negative thoughts and feelings, pregnant women should be advised to seek care from their obstetricians and psychologists, receiving then a better monitoring during the prenatal care, even if remotely^8,19^. When asked about the wish to talk to health professionals during the period of social distancing, 48.9% of the pregnant women indicated that they felt the need to do so. However, fear of exposure to the coronavirus outside the home implied in many women missing their medical appointments, and local health services did not offer options for home visits or remote care^26^.

Most (92.4%) pregnant women said that they had adhered to the social distancing strategy and had stayed at home during the pandemic, reporting that it was a “necessary sacrifice” (88.7%). The social distancing strategy has shown to be one of the best actions to delay the spread of SARS-CoV-2^27^; however, since this measure is gradually being prolonged, the med- and long-term secondary damage caused by social distancing and the symptoms of CMD must be considered in the risk assessment and the care provided to pregnant women^30^.

The selection of the four questions of the Fear of COVID-19 Scale^13^ showed to be suitable for use in Brazil, enabling the verification of differences between groups, and can be used by other national studies. Future studies that follow pregnant women that experienced social distancing and developed CMDs are critical to assess the influence of social distancing on possible obstetric and fetal complications and impaired parental relationships.

Our study has some limitations. First, our data were collected online and thus are not representative of the population of pregnant women, and may particularly underrepresent those who live in conditions of extreme vulnerability and thus do not have access to a smartphone and/or have difficulties filling out the survey. Therefore, our results may provide a best-case scenario of CMD prevalence. Second, we did not use all SRQ questions, since the risk of evoking depressive feelings after reading some questions was considered in a scenario of no feasibility of direct assistance. Moreover, we assessed mental health through questionnaires that are not capable to diagnose mental disease and does not apprehend in depth the feelings and perceptions of the interviewee.

Our novel study showed that, during the period of social distancing, a high prevalence (45.7%) of common mental disorders was observed among pregnant women living in a Brazilian city with a high number of COVID-19 cases. These findings call for the development of public policies and clinical protocols aimed at guaranteeing the health of pregnant women during periods of social distancing.
